# Immune Infertility Should Be Positively Diagnosed Using an Accurate Method by Monitoring the Level of Anti-ACTL7a Antibody

**DOI:** 10.1038/srep22844

**Published:** 2016-03-09

**Authors:** Jun Fu, Rongyan Yao, Yanyun Luo, Dantong Yang, Yang Cao, Yi Qiu, Wei Song, Shiying Miao, Yiqun Gu, Linfang Wang

**Affiliations:** 1 State Key Laboratory of Medical Molecular Biology, Department of Biochemistry and Molecular Biology, Institute of Basic Medical Sciences Chinese Academy of Medical Sciences, School of Basic Medicine Peking Union Medical College, Beijing, People’s Republic of China; 2 Key Laboratory of Birth Regulation and Control Technology of National Health and Family Planning Commission of China, Shandong Provincial Key Laboratory for Improving Birth Outcome Technique, Shandong Provincial Family Planning Institute of Science and Technology, Jinan, People’s Republic of China; 3 National Health and Family Planning Key Laboratory of Male Reproductive Health, National Research Institute for Family Planning, World Health Organization Collaboration Centre for Research in Human Reproduction, Beijing, People’s Republic of China

## Abstract

Infertility is currently a major public health problem. Anti-sperm antibodies (ASAs) markedly reduce sperm quality, which can subsequently lead to male and/or female infertility. The accurate detection of ASAs derived from specific spermatozoa is, therefore, clinically useful. We have focused on the spermatozoa-specific expression protein ACTL7a for many years and have developed an enzyme-linked immunosorbent assay (ELISA) to detect the concentration of anti-ACTL7a antibodies in fertile sera (*n* = 267) and infertile sera (*n* = 193). Infertile sera were collected from the positive sera of tray agglutination tests (TAT), which is a routine ASA screening methodology. We found that the concentration of anti-ACTL7a antibodies was significantly higher in the infertile sera (than in the fertile sera, *P* < 0.0001) and much higher in the TAT ≥ 16 infertile sera. The ELISA was much better for male sera detection (AUC = 0.9899). If we set the standard at a strongly positive value (calculated by ROC curve), the positive predictive value of the antibody detection reached 100 percent, with a false positive rate of zero. The developed ELISA method for anti-ACTL7a antibody detection is therefore sensitive, accurate, and easy to perform, making it an excellent potential tool for future clinical use.

Infertility is a significant public health problem. Couples who fail to conceive after 1 or more years of regular, unprotected sexual intercourse are defined as infertile by the World Health Organization (WHO)^1^,^2^. Anti-sperm antibodies (ASAs) interact with numerous sperm antigens that perform functions in fertility, markedly decreasing the chance of pregnancy by reducing sperm count, motility, and viability, leading to male or female ‘immune infertility’^3^,^4^,^5^,^6^,^7^,^8^,^9^.

Several tests are available to detect ASA in either semen or serum, including ‘direct’ tests for antibodies bound to the sperm and ‘indirect’ tests in sperm-free fluids such as serum. Previously available tests of ASA detection showed only the binding of antibodies on the sperm surface, but no antigenic information could be applied^10^,^11^,^12^. Antibodies derived from accurate, specific spermatozoa antigens should be detected and assessed for possible use in clinical diagnosis.

We have focused on the spermatozoa-specific expression protein ACTL7a for many years. In a previous study, we found that ACTL7a could be recognized by the antibodies contained in infertile serum, which could reduce mouse fertility rates to zero^13^. ACTL7a is located in the acrosome and tail of mature spermatozoa and plays an important role in spermiogenesis^13^,^14^, forming a complex with the cytoskeletal proteins Tes and Mena^15^. We also reported that the active immunization of male or female mice with purified ACTL7a results in a significant reduction in fertility, and the function of ACTL7a during spermiogenesis could be blocked by rabbit anti-ACTL7a antibodies^13^. ACTL7a has been identified as a tyrosine phosphorylation substrate in capacitated buffalo sperm^16^ and is essential for the capacitation of mouse spermatozoa^17^.

In this study, we developed an enzyme-linked immunosorbent assay (ELISA) to accurately detect the concentration of anti-ACTL7a antibodies in hundreds of fertile or infertile human sera collected from 2011 to 2015. Infertile sera were collected from the positive sera of tray agglutination tests (TAT), which are routine screening examinations for anti-sperm antibodies (ASAs)^18^,^19^,^20^. The ELISA we developed here is sensitive, accurate, and easy to perform, making it a potentially valuable tool in future clinical diagnosis.

## Results

### Establishment of an Enzyme-Linked Immunosorbent Assay to Detect Anti-ACTL7a Antibody Concentration in Human Sera

Purified ACTL7a (aa1–70) was used as the antigen in ELISA. Rabbit anti–ACTL7a antibodies, which were diluted at concentrations of 2, 1.5, 1, 0.75, 0.5, 0.25, 0.125, and 0 × 10^−4^ μg/μL, were used as the standard samples. The OD ratios of the standard samples and the concentrations of the antibodies were used to fit a linear regression curve. A mathematical formula (formed as Y = a × X + b; where a and b are two parameters, X is the OD ratio, and Y is the calculated concentration) and variance (R^2^) were calculated. For example, the OD ratios 2.2888, 1.9708, 1.4156, 1.0040, 0.7350, 0.3362, 0.1575 and 0 corresponded to antibody concentrations of 2, 1.5, 1, 0.75, 0.5, 0.25, 0.125, and 0 × 10^−4^ μg/μL. The mathematical formula of the fitting curve for the two group values was Y = 0.8245 × X–0.0494, and the variance (R^2^) was 0.9841. The variance (R^2^) was used to measure the credibility of the tests, and R^2^ over 0.95 was required to determine that a test was successful. The OD ratio of a positive serum (dilution, 1:1000) was 2.237 (X value), and it was calculated to a concentration of 1.80 × 10^−1^ μg/μL (Y value) using the mathematical formula Y = 0.8245 × X-0.0494. A positive control sample in each plate was used to monitor the accuracy of the tests. The OD ratios of human serum samples were calculated for each antibody concentration as described above.

### Immune Infertile Sera Contain Higher Concentrations of Anti-ACTL7a Antibodies

**Figure 1:**
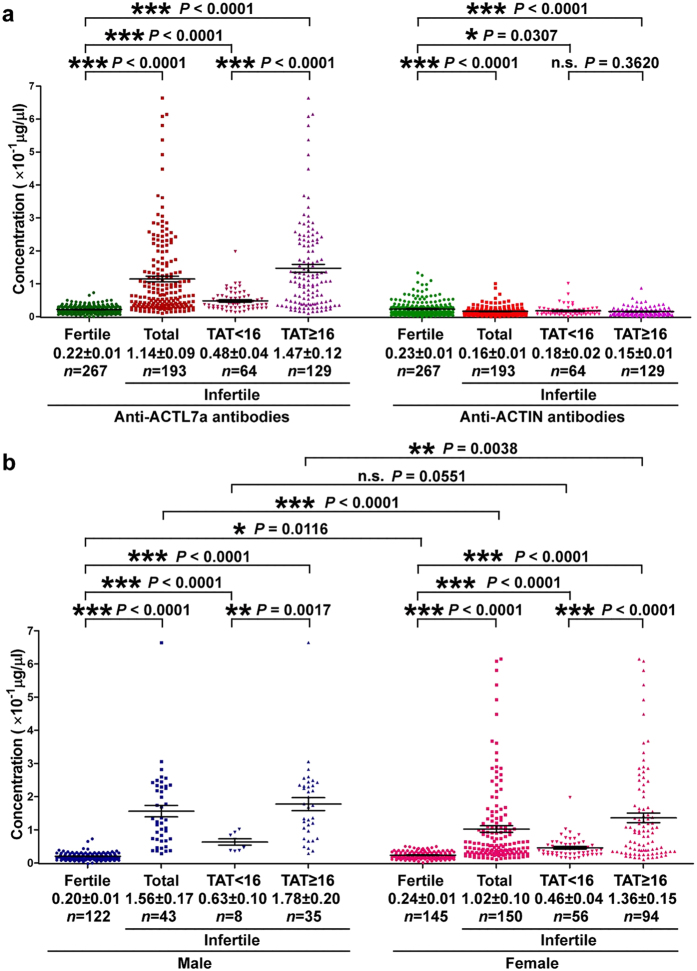
Levels of the concentrations of anti-ACTL7a antibodies or anti-ACTIN antibodies in the sera of the fertile and immune infertile groups (**a**). According to serum titers obtained from TAT tests, the immune infertile sera were further divided into two groups: the TAT < 16 infertile group (titers < 1:16) and the TAT ≥ 16 infertile group (titers ≥ 1:16). (**b**) The levels of the concentrations of anti-ACTL7a antibodies in the sera from the infertile males and females are shown. The concentrations of antibodies are shown as the mean ± SEM. An unpaired *t*-test was performed between male fertile group and female fertile group (*P* = 0.0116); the Mann–Whitney *U*-tests were used to compare the rest statistical tests. ***P < 0.001, **P < 0.01, *P < 0.05, n.s., no significant differences.

Sera were collected from 267 fertile (20–52-year-old subjects, with a mean age of 34.18, fertile parents for paternity tests) and 193 infertile (20–43-year-old subjects, with a mean age of 30.53, fail to conceive after 1 or more years of regular, unprotected sexual intercourse) whose sera were positive for TAT. According to the TAT results, infertile sera were separated into the TAT < 16 group (*n* = 64, 20–41-year-old subjects, with a mean age of 30.34) and TAT ≥ 16 group (*n* = 129, 20–43-year-old subjects, with a mean age of 30.63). Using the ELISA described above, we found that the concentrations of anti-ACTL7a antibodies were significantly higher in the immune infertility group than in the fertile group (*P* < 0.0001, the average concentrations of anti-ACTL7a antibodies were shown as the mean and SEM in Fig. 1a), and the concentrations of antibodies were obviously much higher in the infertile (TAT ≥ 16) group than in the infertile (TAT < 16) group (*P* < 0.0001, more than three times, Fig. 1a). The concentrations of anti-ACTIN antibodies in the fertile and infertile sera were also detected as controls. In contrast to the anti-ACTL7a antibodies, the average concentrations of the anti-ACTIN antibodies declined from 0.23 ± 0.01 × 10^−1^ μg/μL in fertile sera to 0.16 ± 0.01 × 10^−1^ μg/μL in infertile sera (*P* < 0.0001, Fig. 1a). This decline was due to the contribution of the infertile (TAT ≥ 16) group.

### ACTL7a Causes Immune Reactions in Female Infertility Subjects but Induces Much More Stronger Reactions in Male Infertility Subjects

Among the 267 fertile sera, the female sera (*n* = 145) contained higher concentrations of anti-ACTL7a antibodies than the male sera (*n* = 122). The concentration was approximately 20% greater in the female fertile sera than in the male fertile sera (0.24 ± 0.01 × 10^−1^ μg/μL vs. 0.20 ± 0.01 × 10^−1^ μg/μL, respectively; *P* = 0.0116, Fig. 1b). This confirms that the sperm antigen has strong immunogenicity and suggests that ACTL7a is a sperm antigen. However, the average concentrations of the anti-ACTL7a antibodies in the male infertility subjects (*n* = 43) were much higher (approximately 50% higher, *P* < 0.0001, Fig. 1b) than the concentrations in the female infertility subjects (*n* = 150). The significantly higher concentrations of the anti-ACTL7a antibodies were due to the contribution of the TAT ≥ 16 group. The average concentration of the anti-ACTL7a antibodies in the male infertility (TAT ≥ 16) group were much higher than the concentrations in the female infertility (TAT ≥ 16) group (*P* = 0.0038, Fig. 1b). Indeed, the concentration was 1.36 ± 0.15 × 10^−1^ μg/μL in the infertile female subjects (TAT ≥ 16) but 1.78 ± 0.20 × 10^−1^ μg/μL in the infertile male subjects (TAT ≥ 16), suggesting that the sperm antigens induced much more serious reactions in male infertility. There were no significant differences between the male infertile (TAT < 16) group and the female infertile (TAT < 16) group.

### Receiver Operating Characteristic (ROC) Curve Analysis Shows That the Developed ELISA Performs Well for the Detection of Immune Infertilities

**Figure 2:**
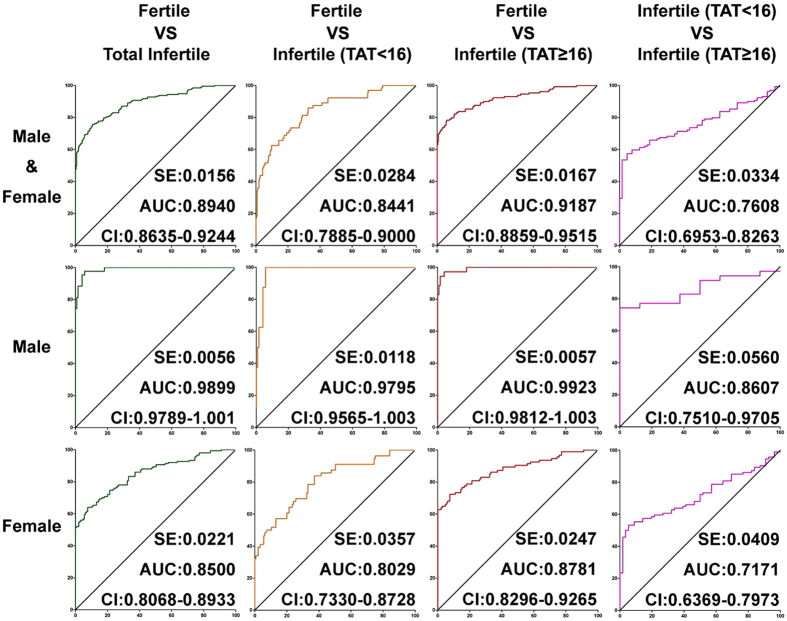
ROC curve analysis of the concentrations of anti-ACTL7a antibodies as measured in the total male and female sera between the fertile group and the total infertile group, the fertile group and the TAT < 16 or TAT ≥ 16 infertile groups, or the two infertile groups. AUC, area; SE, std. error; Cl, confidence interval.

The ROC curve was used to judge the accuracy and sensitivity of this diagnostic approach. As shown in Fig. 2, the area under the ROC curve was 0.8940 for the comparison between the fertile and infertile group. The best cut-off determined by the ROC curves between the fertile group and the infertile group was 0.3449 × 10^−1^ μg/μL, showing that the anti-ACTL7a antibodies in serum with a concentration over 0.3449 × 10^−1^ μg/μL were considered positive according to the ELISA. Using the method described above, the best cut-off determined by ROC curves between the infertile (TAT < 16) group and the infertile (TAT ≥ 16) group was 0.9480 × 10^−1^ μg/μL.

As shown in Fig. 1b, the immune reactions induced by ACTL7a were much different between the male and female subjects. The concentration of anti-ACTL7a was higher in the fertile female group (than in the fertile male group) but significantly higher in the infertile male group. The standards for the male and female subjects should be calculated separately. When comparing the fertile male group with the immune infertile male group, the area under the ROC curve increased to 0.9899. The best cut-off as determined by ROC curves between the fertile male and infertile male groups was 0.3497 × 10^−1^ μg/μL, and this value was defined as a positive point in this literature for antibodies contained in the male serum. The best cut-off as determined by ROC curves between the male infertile (TAT < 16) and male infertile (TAT ≥ 16) groups was 1.0370 × 10^−1^ μg/μL, and this value was defined as a strongly positive point in this literature for antibodies contained in the male sera.

For the comparison between the fertile and immune infertile female groups, the area under the ROC curve was 0.8500. The positive point for females was 0.3854 × 10^−1^ μg/μL and the strongly positive point for females was 0.8838 × 10^−1^ μg/μL, which were both the best cut-off points as determined by the ROC curves.

**Figure 3:**
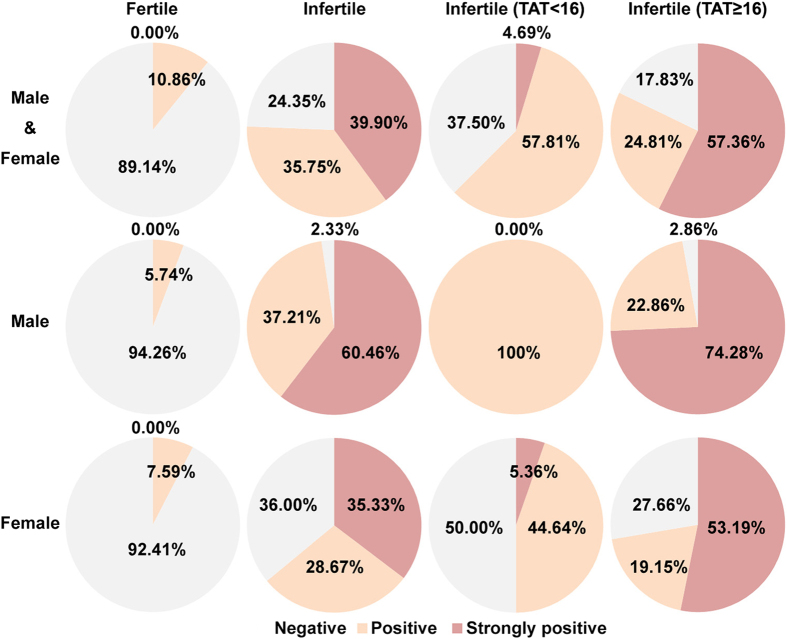
Negative, positive, and strongly positive percentages in the total sera, male sera, and female sera from the fertile group, the total infertile group, the TAT < 16 infertile group, and the TAT ≥ 16 infertile group are shown in grey (negative), red (positive), and dark red (strongly positive).

The percentages of negative, positive and strongly positive results for the fertile and infertile groups, and for the male and female groups are shown in Fig. 3. The percentage of strongly positive male infertility samples was much higher than that for female fertility samples. In the male and female infertile (TAT ≥ 16) groups, strongly positive sera accounted for a large proportion of the samples.

The positive predictive value (PPV), negative predictive value (NPV), true positive rate (TPR), and false positive rate (FPR) for the fertile group and infertile groups were calculated and are shown in Table 1. If we set the standard at a strongly positive value for the comparison between the fertile group and infertile group, the PPV was 100 percent and the FPR zero. Obviously, for the male samples, the PPV, NPV, and TPR were increased and the FPR was decreased. These values suggest that the ELISA method developed here is a viable method for predicting immune infertility, especially in male subjects.

### Primary Infertilities are Associated with Higher Concentrations of Anti-ACTL7a Antibodies than Secondary Infertilities

**Figure 4:**
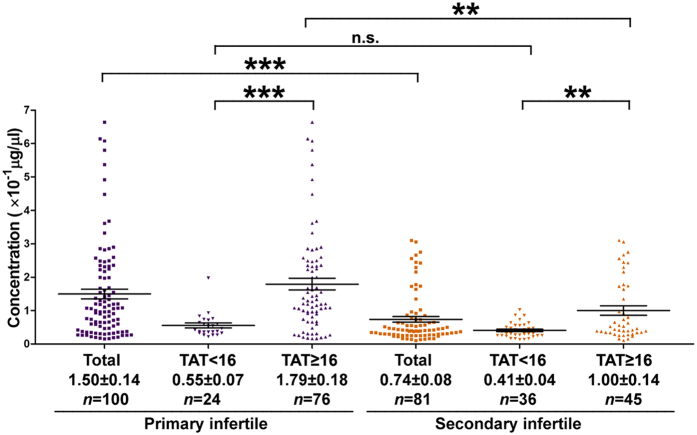
Anti-ACTL7a antibody levels in the sera samples of the primary infertilities and secondary infertilities. Primary infertility is the inability to have one live birth, whereas secondary infertility is the inability to have any additional live births. The concentrations of the antibodies are shown as the mean ± SEM. The Mann–Whitney *U*-tests were used in the comparisons. ***P < 0.001, **P < 0.01, n.s., no significant differences.

Based on previous work, the types of infertility were separated by obstetric history: primary infertility is the inability to have one live birth, whereas secondary infertility is the inability to have any additional live births^21^. We found that the concentrations of anti-ACTL7a antibodies in primary infertilities were two-fold higher than the concentrations in secondary infertilities (*P* < 0.0001, Fig. 4). The significant differences were mainly contributed by TAT ≥ 16 groups (*P* = 0.0018, Fig. 4), and there was no significant difference between the primary infertilities (TAT < 16) and secondary infertilities (TAT < 16).

### The Asthenozoospermia and Azoospermia Male Infertilities Were Positive for Anti-ACTL7a Antibodies

Of the male infertility samples studied, seventeen were subjected to an analysis of semen quality. Six patients suffered from asthenozoospermia, and six patients suffered from Azoospermia (non-obstructive). All twelve patients were primary infertility patients. The average concentration of anti-ACTL7a antibodies in the six asthenozoospermia subjects was 1.4800 ± 0.2332 μg/μL, which was much higher than for the fertile males, and all six of the asthenozoospermia sera (100%) were positive and five (83.33%) were strongly positive. The average concentrations of the anti-ACTL7a antibodies in the six Azoospermia were 0.6768 ± 0.0644 μg/μL, and all of them (100%) were positive. Three of the Azoospermia subjects had small testes, and another had normal testes. There were no differences in the concentration of anti-ACTL7a antibodies between the small testes and normal testes subjects.

## Discussion

Specific sperm antigens should be used to detect immune infertilities. ACTL7a is expressed in male germ cells from the spermatid to spermatozoa, after meiosis of the spermatocyte^13^. The spermatids are separated from the spermatogania and preleptotene spermatocytes by the blood-testes barrier and are not involved in monitoring the immune system^22^,^23^. ACTL7a has immune antigenicity and causes fertile women to produce slightly higher concentrations of anti-ACTL7a antibodies than fertile men. However, ACTL7a was found at significantly higher concentrations in male infertilities than in female infertilities. When antigens against the universal expression protein ACTIN were detected in the sera, as opposed to the spermatozoa-specific expression protein ACTL7a, they were found to be higher in the fertile sera than in the infertile sera. Compared to the infertile group, some sera had higher concentrations of anti-ACTIN antibodies in the fertile group, which suggests that these false positive results may influence the diagnosis of infertility.

Negative results were found for nearly all of the groups (including the male and female infertility groups). Although the sera collected from the infertile (TAT ≥ 16) group had higher levels of TAT, approximately one-sixth of the subjects tested negative for the anti-ACTL7a antibody based on ELISA; the female infertilities (TAT ≥ 16) contributed greatly to the negative results. Including TAT, the various available ASA detections could not supply the antigenic information. If the antibody to some universal protein on the surface of spermatozoa reaches a high enough titer, it will show up as positive in the TAT tests. Spermatozoa agglutination can also be caused by other ASAs. We suggest that accurate antigenic information should be obtained in clinical diagnosis.

The AUC, which can be calculated using a ROC curve, is a good measurement of the performance of a diagnostic test^24^,^25^. The closer the value is to 1, the better its performance on a diagnostic test. The AUC calculated by the ROC curve between the fertile and infertile groups was 0.8940, which suggests that the ELISA developed here performed well. For the male subjects, the AUC was 0.9899, as calculated between the fertile and infertile male groups (Fig. 2). The fact that the AUC (0.9899) was so close to 1 illustrates that the ELISA detection of the anti-ACTL7a antibodies worked well for predicting male infertilities. The PPV, NPV, TPR, and FPR values are used as performance measurements for diagnostic tests^26^, and these values indicate that the ELISA worked very well for the detection of antibodies in the male sera (Table 1). If we set the cut-off at a strongly positive value, the PPV for the males and females reached 100%, and the FPR decreased to zero (Table 1), indicating that the ELISA developed here for detection of anti-ACTL7a antibodies in the sera is a good indicator of fertility.

The main causes for primary infertility are low semen quality^27^ and ovulatory disorders^1^. However, the causes for secondary infertilities mixed with multiple factors include reproductive tract infections, caesarean scar syndrome, previous pelvic surgery, and sexual dysfunction^28^,^29^,^30^,^31^,^32^,^33^. The sera from male infertilities and primary infertilities contained higher concentrations of anti-ACTL7a antibodies, indicating that male infertilities and primary infertilities containing higher ASAs should be paid more attention and would be better treated as immune infertilities.

ASAs may reduce sperm count, motility, and viability, which lead to a corresponding reduction in fertility^6^,^32^,^34^. Among the male infertilities, six patients suffered from asthenozoospermia and six patients suffered from Azoospermia. All twelve sera from the male primary infertilities had positive results in the ELISA. Most of the asthenozoospermia (five out of six) received strongly positive results. The high concentrations of anti-ACTL7a antibodies in the asthenozoospermia or Azoospermia cases reflect the deleterious effects of ASAs on spermatozoa.

In conclusion, we developed an ELISA to determine the accurate concentrations of anti-ACTL7a antibodies in human sera. A very small amount of serum was used in the ELISA for one sample, and the detection of anti-ACTL7a antibodies could be performed with other serologic tests. The materials needed for our ELISA method are stable, ensuring that the obtained results are reliable and reproducible. Future studies should focus on additional antigens related to fertility that can induce ASAs, which may aid in the diagnosis and treatment of infertility.

## Methods

### Ethics Statement

All experimental protocols were approved by the institutional review board of the Institute of Basic Medical Sciences of the Chinese Academy of Medical Sciences (designated as 040-2013). All experiments were performed in accordance with relevant guidelines and regulations. The sera used in this manuscript were residual collected from clinical examination. Very litter serum (less than 5 μL) was used for one sample, and we don’t take the blood again. The sera were numbered after collection, so the researchers could not see the name of the patients. The result for one sample was presented as a dot in the figures. According to the protocols above, the institutional review board of the Institute of Basic Medical Sciences of the Chinese Academy of Medical Sciences approved exemption of informed consent.

### Collection of Sera from Infertile and Fertile Subjects

Sera collected from infertile or fertile individuals were subjected to TAT tests according to the WHO guidelines. Sample information, such as the spermatozoa parameters for male infertility or endocrine index and gynaecology examination for female infertility, was recorded if infertile patients were examined. Following the WHO guidelines (2010), azoospermia was defined as absence of sperm cells in semen; asthenozoospermia was defined as WHO grade a + b, with sperm motility < 50%, or WHO grade a < 25%^35^; Oligospermia was defined as having a sperm concentration of < 20 million/ml^36^.

### Enzyme-Linked Immunosorbent Assay

The ACTL7a (1-70 aa) protein was purified as previously described. The purified protein (antigen) was coated in wells overnight at 4 °C. The wells were washed and blocked with bovine serum albumin and incubated overnight at 4 °C with fertile sera, infertile sera, and positive sera (at a dilution of 1:1000), or rabbit anti–ACTL7a antibody (at concentrations of 2, 1.5, 1, 0.75, 0.5, 0.25, 0.125, and 0 × 10^−4^ μg/μL for the standard curve). The plates were washed and incubated with horseradish peroxidase–conjugated reagent (Thermo Scientific #21230, USA; the reagent can identify both human and rabbit immunoglobulin G) for 2 hours at 37 °C. The wells were washed and incubated with substrate solution. Finally, HCl was added to each well and monitored at a wavelength of 490 nm using a microplate reader (Multiscan GO, Thermo Scientific, USA). The detection of anti-ACTIN antibody was performed as described above, but using ACTIN (sigma) as the coated antigen and using rabbit-anti-ACTIN antibody to generate the standard curve.

### Statistical Analysis

The data are reported as the mean and SEM. All statistical analyses were performed using Prism version 5.0 for Windows. First, the data were analysed for the normality of distribution using the Kolmogorov–Smirnov normality test (*n* > 50) or the Shapiro–Wilk normality test (*n* ≤ 50). Secondly, an unpaired *t*-test was performed on the normality distribution and the variance similar population, and the Mann–Whitney *U*-test was used to compare the groups derived from the stratified or the variance heterogeneity population. *P* < 0.05 (two-tailed) was considered significant. *P* values are provided in the figures and text of the manuscript at the appropriate places; however, Prism version 5.0 does not report actual values when *P* < 0.0001. *P* < 0.05 was marked as ‘*’*P* < 0.01 was marked as ‘**’ and *P* < 0.001 was marked as ‘***’.

## Additional Information

**How to cite this article**: Fu, J. *et al.* Immune Infertility Should Be Positively Diagnosed Using an Accurate Method by Monitoring the Level of Anti-ACTL7a Antibody. *Sci. Rep.* **6**, 22844; doi: 10.1038/srep22844 (2016).

## Figures and Tables

**Table 1:** Positive predictive value (PPV), negative predictive value (NPV), true positive rate (TPR), and false positive rate (FPR) as calculated for the fertile group and total infertile group.

Group	PPV (%)	NPV (%)	TPR (%)	FPR (%)
Antibodies > 0.3449 × 10^−1^ μg/μL	83.43	83.51	75.65	10.86
Antibodies > 0.9480 × 10^-1^ μg/μL	100	69.71	39.90	0
Male				
Antibodies > 0.3497 × 10^−1^ μg/μL	85.71	99.14	97.67	5.74
Antibodies > 1.0370 × 10^−1^ μg/μL	100	87.77	60.46	0
Female				
Antibodies > 0.3854 × 10^−1^ μg/μL	89.72	71.28	64.00	7.59
Antibodies > 0.8838 × 10^−1^ μg/μL	100	59.92	35.33	0
